# Modified biliary-enteric anastomosis for congenital choledochal cyst: clinical and prognostic analysis of 91 cases

**DOI:** 10.1007/s00383-017-4077-4

**Published:** 2017-03-13

**Authors:** Ji Chen, Bin Jiang, Jun Yi, Lei Huang, Xinmin Si

**Affiliations:** 1 Department of General Surgery, Children’s Hospital of Nanjing Medical University, Nanjing, 210008 China; 2 Department of Gastroenterology, The First Affiliated Hospital of Nanjing Medical University, No.300 of Guangzhou Road, Nanjing, 210029 China

**Keywords:** Hepaticojejunostomy, Choledochal cyst, Pediatrics, Follow-up

## Abstract

**Purpose:**

To report our experience with a modified biliary-enteric anastomosis procedure for the surgical treatment of congenital choledochal cysts.

**Methods:**

Between January 2009 and December 2013, 91 children (19 boys, 72 girls; ages, 6–145 months) with congenital choledochal cysts were treated with our modified surgical procedure in our hospital. Of these patients, 69 had type I cysts, and 22 had type IV B cysts. The main parameters analyzed mainly included the operative time, duration of bowel recovery, resumption of diet, postoperative hospital stay, liver-function tests, postoperative complications, and prognosis.

**Results:**

The average operation duration was 129.34 ± 23.50 min. The time until first flatus and resumption of oral diet were 26.51 ± 4.13 h and 5.47 ± 0.77 day, respectively. The mean postoperative hospital stay was 11.84 ± 2.58 day. Postoperative complications occurred in six patients: intestinal obstruction (1 patient), postoperative bleeding (1 patient), postoperative pancreatitis (1 patient), and bile leakage (3 patients). During a follow-up of 2–7 years, four cases of occasional abdominal pain were found. Contrast agent reflux was detected on upper gastrointestinal imaging in three children. All children had good nutrition.

**Conclusion:**

The modified biliary-enteric anastomosis is a safe, simple, and reliable technique. However, longer follow-up and a larger sample size are necessary to prove its efficacy in the treatment of congenital choledochal cysts.

## Introduction

Congenital choledochal cysts (CCs) are congenital anomalies of the biliary ducts characterized by cystic dilatation of the extra- and/or intrahepatic biliary ducts [1, 2]. Untreated CCs are associated with complications such as recurrent cholangitis, acute pancreatitis, and cholangiocarcinoma [3–5]. In the past, CCs were primarily treated using simple internal drainage via cystenterostomy or partial cyst excision. However, this treatment was associated with adverse clinical outcomes, such as stomal stenosis, cholestasis, cholangiolithiasis, and even cholangiocarcinoma [6, 7], resulting in poor prognosis and even secondary surgical operation. At present, the surgical approach to CC depends on the clinical cyst type as defined in the Todani classification system [8]. The standard procedure for type I and IVB CCs is complete resection of the cyst followed by a Roux-en-Y hepaticojejunostomy [9, 10]. However, on the basis of our experience, we have been performing a modified biliary-enteric anastomosis, also called an “uncut” anastomosis, after CC excision in our center. The present single-center, retrospective analysis was performed to determine the effectiveness of our surgical protocol and report the prognosis in 91 CC patients treated with this technique.

## Materials and methods

The study protocol was approved by the institutional review board of the Children’s Hospital of Nanjing Medical University in accordance with guidelines. Informed consent was obtained from the parents of all patients.

### Inclusion criteria

Children aged between 0 and 14 years and were admitted to the Department of General Surgery, Children’s Hospital of Nanjing Medical University from January 2009 to December 2013. The diagnosis of CC was confirmed using ultrasonography, abdominal computed tomography (CT), and magnetic resonance cholangiopancreatography. Type I and IVB CC were enrolled; other types were excluded. This study ultimately involved 91 children who underwent CC resection and modified biliary-enteric anastomosis.

### Preoperative preparation

Biliary infection was treated before operation. Children with prolonged prothrombin time secondary to cholestasis were corrected with intravenous vitamin K.

### Surgical technique

**Fig. 1:**
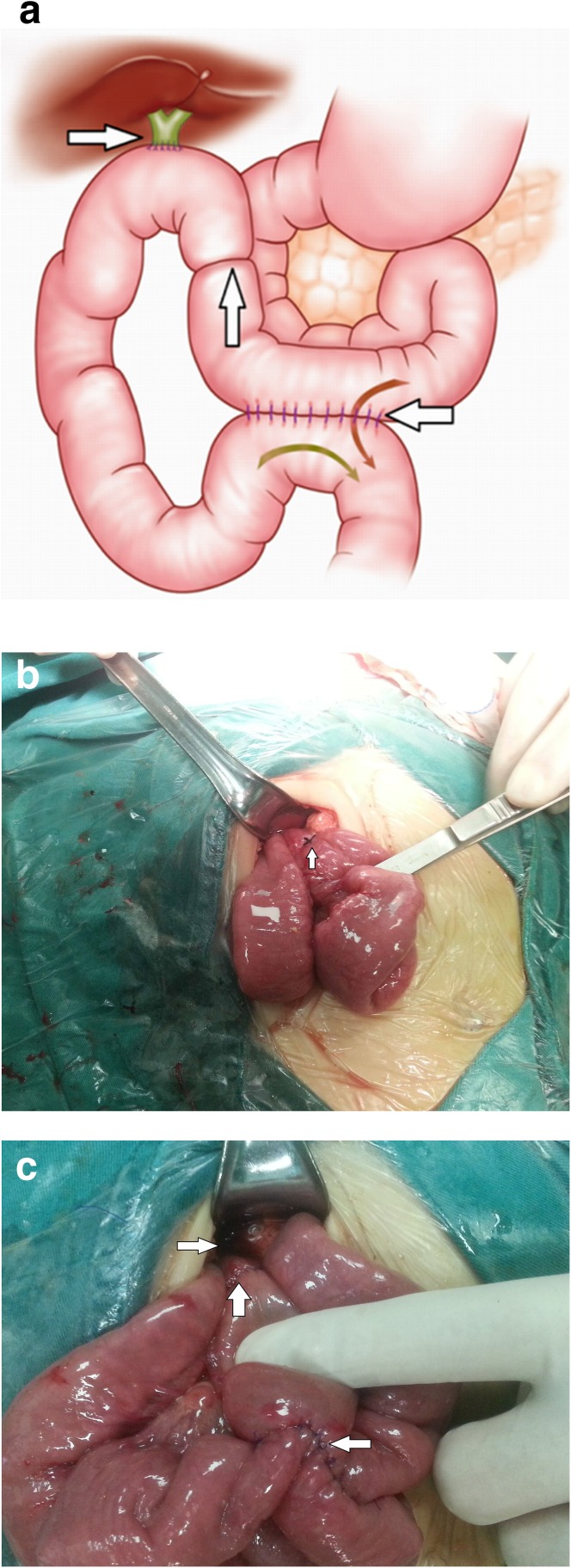
The modified biliary-enteric (Warren) anastomosis: **a** diagrammatic representation and **b, c** intraoperative photograph showing the biliary-enteric anastomosis (*right arrow*), jejunojejunostomy (*left arrow*), and jejunal occlusion (*up arrow*). The jejunojejunostomy is located 25 cm from the biliary-enteric anastomosis, and the jejunal occlusion is located 2 cm from the biliary-enteric anastomosis

With the patient in a supine position after the combined intravenous-inhalation anesthesia, the abdomen was entered through an approximately 5-cm oblique incision below the costal margin in the right upper quadrant. The cystic artery and duct were identified, and the gallbladder was mobilized from the liver bed, so that it was free except for the cystic artery and duct. The cystic artery was ligated and divided. After the complete resection of the cyst, we raised the jejunum about 25-cm distal to the ligament of Treitz and made an end-to-side anastomosis of the bile duct with the jejunum in front of the transverse colon. The afferent loop was ligated with 1/0 silk suture and reinforced with interrupted seromuscular sutures 2-cm distal to the biliary-enteric anastomosis. Lastly, a side-to-side jejunojejunostomy was made; the afferent loop was about 10 cm long, and the efferent loop was about 25 cm long (Fig. 1).

### Postoperative care

Oral feeding was initiated after the fluid from the gastric tube became clear, usually by postoperative day 5 or 7. The abdominal drain was removed on day 5 if there is no evidence of leak from the biliary-enteric anastomosis.

### Follow-up

The patients were followed up at 6, 12, 24, 48, and 84 months after operation. We mainly focused on the patients’ clinical symptoms and abdominal signs. CT, ultrasonography, endoscopy, and liver-function tests were performed according to patients positive symptoms. Upper gastrointestinal imaging was performed once every two years.

### Analyzed parameters

The main parameters analyzed mainly included the operative time, duration of bowel recovery, resumption of diet, postoperative hospital stay, liver-function tests, postoperative complications and prognosis.

### Statistical analysis

Data were expressed as mean ± standard error of the mean. Statistical analyses were performed using the Statistical Package for the Social Sciences software 21.0 software (SPSS, Inc., Chicago, IL, USA), and the Student *t*-test. *P* < 0.05 was deemed to be statistically significant.

## Results

### General patient characteristics

Of the 91 study patients, 19 were boys and 72 were girls. The mean aged was 42.42 ± 32.61 months (ranged from 6 to 145 months). In total, 69 patients had type I CCs, and 22 patients had type IVB CCs. The clinical manifestations included abdominal pain, vomiting, jaundice, white stools, and fever.

### Operation time and postoperative recovery

The mean operation time was 129.34 ± 23.50 min. The time until recovery of bowel peristalsis was 26.51 ± 4.13 h, while the time until resumption of diet was 5.47 ± 0.77 day. The mean postoperative stay was 11.84 ± 2.58 day. None of these parameters significantly differed between type I and type IVB CC patients (Table 1).

**Table 1:** Perioperative observation items of modified group versus Roux-en-Y group

	Modified group (*n* = 91)	Roux-en-Y group (*n* = 62)	*P*-value
Operative time: min	129.34 ± 23.50	200.24 ± 87.04	*P* < 0.001
Intraoperative blood loss: mL	9.12 ± 4.55	16.21 ± 7.80	*P* < 0.001
Duration of bowel recovery: h	26.51 ± 4.13	48.21 ± 4.81	*P* < 0.001
Resumption of diet: days	5.47 ± 0.77	6.95 ± 1.27	*P* = 0.022
Duration of drainage: h	59.12 ± 16.33	71.14 ± 14.52	*P* < 0.001
Postoperative hospital stay:days	11.84 ± 2.58	12.90 ± 3.82	*P* = 0.013

### Liver-function tests

Among the 24 children who had normal blood biochemical parameters preoperatively, only one child showed slightly elevated levels of alanine transaminase (ALT) and aspartate transaminase (AST) postoperatively. In the 67 patients with abnormal biochemical indexes before the surgery, we rechecked the parameters on the fifth, seventh, and tenth day after the surgery. The results are shown in Table 2.

**Table 2:** The recovery of biochemical indexes after operation

Abnormal index	Abnormal cases (before surgery)	Abnormal cases (5th day)	Abnormal cases (7th day)	Abnormal cases (10th day)	Average recovery days
ALT	47	10	6	3	4.88
AST	52	17	11	4	5.27
GGT	62	54	34	9	7.63
TBIL	34	12	8	3	5.03

### Complications

Six patients developed complications such as postoperative bleeding, pancreatitis, and bile leakage. Postoperative bleeding and pancreatitis occurred in one patient (1.1%) each and resolved after conservative therapy. Bile leakage occurred in three patients (3.3%), all of whom were treated with sufficient drainage. Bowel obstruction occurred in one patient (1.1%) and required reoperation. We found that the bile loop had twisted by 180° in this patient; we therefore reanastomosed the bile duct with the jejunum.

### Follow-up

In total, 70 patients (76.9%) attended follow-up for 2–7 years. Four children complained of occasional abdominal pain in the 15th, 22th, 33th, and 58th month after surgery, respectively, but organic illnesses were ruled out in these patients on CT, ultrasonography, and endoscopy. Upper gastrointestinal imaging was performed once every two years; three children showed contrast agent reflux but were clinically asymptomatic. In the other children, gastrointestinal imaging was normal. All the children had good nutrition.

## Discussion

Biliary-enteric anastomosis was first described by Warren in 1967 [11]. This anastomosis preserves the myoneural continuity between the afferent and efferent loops but does not prevent the passage of food into the bile duct loop. The technique was therefore modified by making a luminal occlusion in the afferent limb distal to the hepaticojejunostomy stoma. We designed a modified biliary-enteric anastomosis procedure by including ligature of the afferent loop with a silk suture. We hypothesized that this modified technique had three advantages over conventional Roux-en-Y hepaticojejunostomy. First, the modified biliary-enteric anastomosis can be performed without transection of the jejunum; it thus avoids separation of the duodenal pacemaker from the jejunum, maintains the integrity of the jejunum, and allows normal conduction of myenteric impulses [12]. Huang et al. have proved that the silk suture placed across the jejunal wall in the modified Warren operation does not disrupt myoelectrical continuity in the uncut bile duct-enteric loop in rabbits. Ectopic pacemakers did not appear in the limb, and bile duct-enteric loop transition was preserved [13]. Second, the myoelectrical continuity of the jejunum helped maintain peristalsis in the bile duct-enteric loop, preventing food and bile stasis in the anastomotic limb. Park et al. performed the uncut Roux-en-Y reconstruction after laparoscopic distal gastrectomy, and found that the amount of residual gastric contents was significantly lower in the uncut group than in the Roux-en-Y group [14]. Third, the modified biliary-enteric anastomosis procedure is simpler than the conventional Roux-en-Y anastomosis and can be performed in elderly as well as pediatric patients.

Considering these advantages, we have applied this modified technique for the treatment of congenital CCs in our hospital since 2009. In the current study, all the children recovered completely and had a normal quality of life. Abnormal liver function, if present preoperatively, recovered after the operation. High gamma glutamyl transferase (GGT) levels before the operation significantly decreased after the operation. Additionally, the total bilirubin level recovered well after the operation, indicating that bile could smoothly pass through the previously inflamed or obstructed biliary tract. Postoperative complications occurred in six patients and consisted of hemorrhage, bile leakage, bowel obstruction, and postoperative pancreatitis. Exploratory laparotomy was performed in the patient with bowel obstruction; the bile loop had twisted 180° in this patient, and we therefore reanastomosed the bile duct with the jejunum. Bile leakage was managed with peritoneal drainage. Postoperative pancreatitis resolved after fasting, acid suppression, enzyme inhibition, and anti-inflammatory drug administration for 2 weeks. Postoperative bleeding was treated with transfusion, hemostasis, and fluid infusion.

During follow-up, postprandial abdominal pain occurred in four children (5.7%). In all four children, organic illnesses were ruled out by CT, ultrasonography, and endoscopy. All children had good nutrition according to the nutritional assessment. The liver-function tests were normal during the follow-up period.

Food reflux into the bile duct can cause reflux cholangitis, and might increase the risk of cholangiocarcinoma and reduce the quality of life. Although the exact mechanisms for cancer development are not yet fully understood, many investigators have asserted that inflammatory stimulation is related to cholangiocarcinoma development [3, 15, 16]. Furthermore, food or bile reflux can cause clinical symptoms such as abdominal pain, nausea, and vomiting [10, 17]. Of the 65 patients who underwent upper gastrointestinal imaging, 3 had slight contrast agent reflux, yielding a reflux incidence rate of 4.62%. However, these three children had a normal quality of life. The four children with postprandial abdominal pain had normal upper gastrointestinal imaging. We found that the contrast agent refluxed into the bile duct quickly flowed out into the distal jejunum. As mentioned above, the myoelectrical continuity of the jejunum helped maintain peristalsis in the bile limb, preventing the stasis of food and bile in the efferent loop.

The modified biliary-enteric anastomosis is known to reduce food or bile stasis by maintaining the integrity of the intestinal canal and the normal conduction of myenteric impulses [12, 18]. However, because of the possibility of silk suture loose, many authors considered that this technique was associated with a high risk of recanalization of the jejunum [19, 20]. In addition, tight silk sutures can lead to ischemic necrosis of the bowel. In our study, the standard for the tightness of the silk suture was to leave exactly enough space for the tip of a mosquito forceps to pass between the suture and the intestinal wall. Long-term follow-up is required to determine the incidence of recanalization after the surgery.

## Conclusion

This study shows that the modified biliary-enteric anastomosis procedure is a safe, simple, and reliable technique with few intra- and postoperative complications. However, long-term follow-up and a larger sample size are necessary to prove the efficacy of this technique for congenital CCs.

## References

[1] Soares KC, Arnaoutakis DJ, Kamel I, Rastegar N, Anders R, Maithel S, Pawlik TM (2014). Choledochal cysts: presentation, clinical differentiation, and management. J Am Coll Surg.

[2] Moslim MA, Takahashi H, Seifarth FG, Walsh RM, Morris-Stiff G (2016). Choledochal Cyst Disease in a Western Center: a 30-year experience. J Gastrointest Surg.

[3] Soares KC, Kim Y, Spolverato G, Maithel S, Bauer TW, Marques H, Sobral M, Knoblich M, Tran T, Aldrighetti L, Jabbour N, Poultsides GA, Gamblin TC, Pawlik TM (2015). Presentation and clinical outcomes of choledochal cysts in children and adults: a multi-institutional analysis. JAMA Surg.

[4] Muthucumaru M, Ljuhar D, Panabokke G, Paul E, Nataraja R, Ferguson P, Dagia C, Clarnette T, King S (2016). Acute pancreatitis complicating choledochal cysts in children. Acute pancreatitis complicating choledochal cysts in children. J Paediatr Child Health.

[5] Shimada T, Sakata J, Ando T, Yuza K, Toge K, Hirose Y, Katada T, Ishikawa H, Miura K, Ohashi T, Takizawa K, Takano K, Kobayashi T, Tomita H, Wakai T (2016). Resection for carcinoma arising from the remnant intrapancreatic bile duct after excision of a congenital choledochal cyst—a case report. Gan Kagaku Ryoho.

[6] Watanabe Y, Toki A, Todani T (1999). Bile duct cancer developed after cyst excision for choledochal cyst. J Hepatobiliary Pancreat Surg.

[7] Singham J, Yoshida EM, Scudamore CH (2010). Choledochal cysts. Part 3 of 3: management. Can J Surg.

[8] Todani T, Watanabe Y, Narusue M, Tabuchi K, Okajima K (1977). Congenital bile duct cyst: classification, operative procedure, and review of 37 cases including cancer arising from choledochal cyst. Am J Surg.

[9] Lu B, Shen Z, Yu J, Yang J, Tang H, Ma H (2015). Laparoscopic surgery for removal of choledochal cysts and Roux-en-Y anastomosis. Int J Clin Exp Med.

[10] Qiao G, Li L, Li S, Tang S, Wang B, Xi H, Gao Z, Sun Q (2015). Laparoscopic cyst excision and Roux-Y hepaticojejunostomy for children with choledochal cysts in China: a multicenter study. Surg Endosc.

[11] Warren KW (1965). Modification of the Roux-en-Y procedure. Surg Clin N Am.

[12] Kiciak A, Woliñski J, Borycka K, Zabielski R, Bielecki K (2007). Roux-en-Y or ‘uncut’ Roux procedure? Relation of intestinal migrating motor complex recovery to the preservation of the network of interstitial cells of Cajal in pigs. Exp Physiol.

[13] Huang L, Liang LJ, Lai JM (2008). Determination of the postoperative effect on intestinal structure and myoelectric motility in rabbits: modified uncut jejunal loop versus Roux-en-Y biliodigestive anastomosis. Zhonghua Wai Ke Za Zhi.

[14] Park JY, Kim YJ (2014). Uncut Roux-en-Y reconstruction after laparoscopic distal gastrectomy can be a favorable method in terms of gastritis, bile reflux, and gastric residue. J Gastric Cancer.

[15] Funabiki T, Matsubara T, Miyakawa S, Ishihara S (2009). Pancreaticobiliary maljunction and carcinogenesis to biliary and pancreatic malignancy. Langenbecks Arch Surg.

[16] Sastry AV, Abbadessa B, Wayne MG, Steele JG, Cooperman AM (2015). What is the incidence of biliary carcinoma in choledochal cysts, when do they develop, and how should it affect management?. World J Surg.

[17] Yeung F, Chung PH, Wong KK, Tam PK (2015). Biliary-enteric reconstruction with hepaticoduodenostomy following laparoscopic excision of choledochal cyst is associated with better postoperative outcomes: a single-centre experience. Pediatr Surg Int.

[18] Tu BN, Kelly KA (1995). Elimination of the Roux stasis syndrome using a new type of “uncut Roux” limb. Am J Surg.

[19] Mulholland MW, Magallanes F, Quigley TM, Delaney JP (1983). In-continuity gastrointestinal stapling. Dis Colon Rectum.

[20] Tu BN, Sarr MG, Kelly KA (1995). Early clinical results with the uncut Roux reconstruction after gastrectomy: limitations of the stapling technique. Am J Surg.

